# Crystal structure and Hirshfeld surface analysis of 2-amino-3-hy­droxy­pyridin-1-ium 6-methyl-2,2,4-trioxo-2*H*,4*H*-1,2,3-oxa­thia­zin-3-ide

**DOI:** 10.1107/S2056989020003813

**Published:** 2020-03-27

**Authors:** Sevgi Kansiz, Md. Serajul Haque Faizi, Tansu Merve Aydin, Necmi Dege, Hasan Icbudak, Irina A. Golenya

**Affiliations:** a Department of Fundamental Sciences, Faculty of Engineering, Samsun University, Samsun, 55420, Turkey; b PG Department of Chemistry, Langat Singh College, B. R. A. Bihar University, Muzaffarpur, Bihar-842001, India; c Department of Chemistry, Faculty of Arts and Sciences, Ondokuz Mayıs, University, Samsun, 55200, Turkey; d Department of Physics, Faculty of Arts and Sciences, Ondokuz Mayıs, University, Samsun, 55200, Turkey; e Department of Chemistry, Volodymyrska str., 64, National Taras Shevchenko University, 01601 Kyiv, Ukraine

**Keywords:** crystal structure, acesulfame, 2-amino-3-hy­droxy­pyridine, hydrogen bonding, Hirshfeld surface analysis

## Abstract

The asymmetric unit of the title compound, C_5_H_7_N_2_O^+^·C_4_H_4_NO_4_S^−^, contains one cation and one anion. The 6-methyl-2,2,4-trioxo-2*H*,4*H*-1,2,3-oxa­thia­zin-3-ide anion adopts an envelope conformation with the S atom as the flap. In the crystal, the anions and cations are held together by N—H⋯O, N—H⋯N, O—H⋯O and C—H⋯O hydrogen bonds, thus forming a three-dimensional structure. The Hirshfeld surface analysis and fingerprint plots reveal that the crystal packing is dominated by O⋯H/H⋯O (43.1%) and H⋯H (24.2%) contacts.

## Abstract 2

In the crystal of the title compound, C_9_H_11_N_3_O_5_, the 2-amino-6-hy­droxy­pyridin-1-ium cations and 6-methyl-2,2,4-trioxo-2*H*,4*H*-1,2,3-oxa­thia­zin-3-ide anions are held together through N—H⋯O, N—H⋯N, O—H⋯O and C—H⋯O hydrogen bonds.

## Chemical context   

Food additives are substances added intentionally to foodstuffs to perform certain functions such as to impart colour, to sweeten or preserve. They play an essential role in the modern food industry, supporting quality and safety. In this context, artificial sweeteners are widely used in food, beverage, confectionery and pharmaceutical products throughout the world (Clauss & Jensen, 1973 ▸; Ni *et al.*, 2009 ▸). Oxa­thia­zinone dioxide, systematic name 6-methyl-1,2,3-oxa­thia­zin- 4(3*H*)-one 2,2-dioxide and also known as 6-methyl-3,4-di­hydro-1,2,3- oxa­thia­zin-4-one 2,2-dioxide or acesulfame, has been widely used as a non-caloric artificial sweetener (Duffy & Anderson, 1998 ▸) since 1988, after the FDA (US Food and Drug Administration) granted approval. Many countries have approved the use of acesulfame-K in soft drinks, toothpaste, candies, mouthwash, cosmetics and pharmacological preparations (Mukherjee & Chakrabarti, 1997 ▸). The chemistry of acesulfame is of inter­est not only because of its biological importance but also in relation to its coordination properties, since the acesulfame anion offers different donor atoms to metal ions, namely the imino nitro­gen, ring oxygen, one carbonyl and two sulfonyl oxygen atoms. To advance the knowledge of such compounds, we report the synthesis, single crystal structure determination and Hirshfeld surface analysis of the 2-amino-3-hy­droxy­pyridinium acesulfamate salt (I).

## Structural commentary   

**Figure 1:**
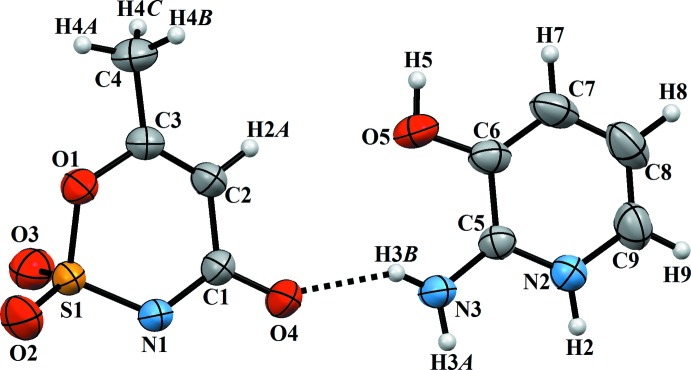
A view of the asymmetric unit of the title compound with the atom-numbering scheme. Displacement ellipsoids are drawn at the 50% probability level. The N—H⋯O hydrogen bond is shown as a dashed line.

A view of the asymmetric unit of (I) with the atom-numbering scheme is shown in Fig. 1 ▸. In the acesulfamate anion, the bond dimensions correspond to the given structural formula with double bonds C1=O4 and C2=C3 and a single bond C1—C2 (Table 1 ▸). A relatively short N1—C1 bond indicates strong π-conjugation in the N1—C1=O4 fragment. Overall, the bond lengths in this anion compare well with those observed in other acesulfamate salts known from the literature (Yıldırım *et al.*, 2019 ▸; Kansız *et al.*, 2019 ▸). The six-membered acesulfamate ring adopts an envelope conformation with atom S1 as the flap; its deviation from the basal plane is 0.555 (1) Å. The basal plane of the envelope is slightly twisted, with an O1—C3—C1—N1 torsion angle of 2.2 (2)°. The cyclic bond lengths in the 2-amino-3-hy­droxy­pyridinium cation agree well with its aromatic nature. The short N3—C5 distance indicates strong conjugation of the amino N3 atom with the acceptor π-system of the pyridinium ring. The cation is almost planar, the largest deviation from the least-squares plane of 0.008 (2) Å is observed for atom C6. The least-squares planes through the cation and the basal atoms of anion form a dihedral angle of 6.47 (11)°.
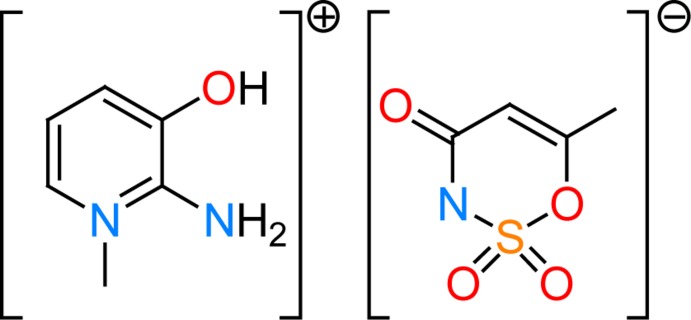


## Supra­molecular features   

**Figure 2:**
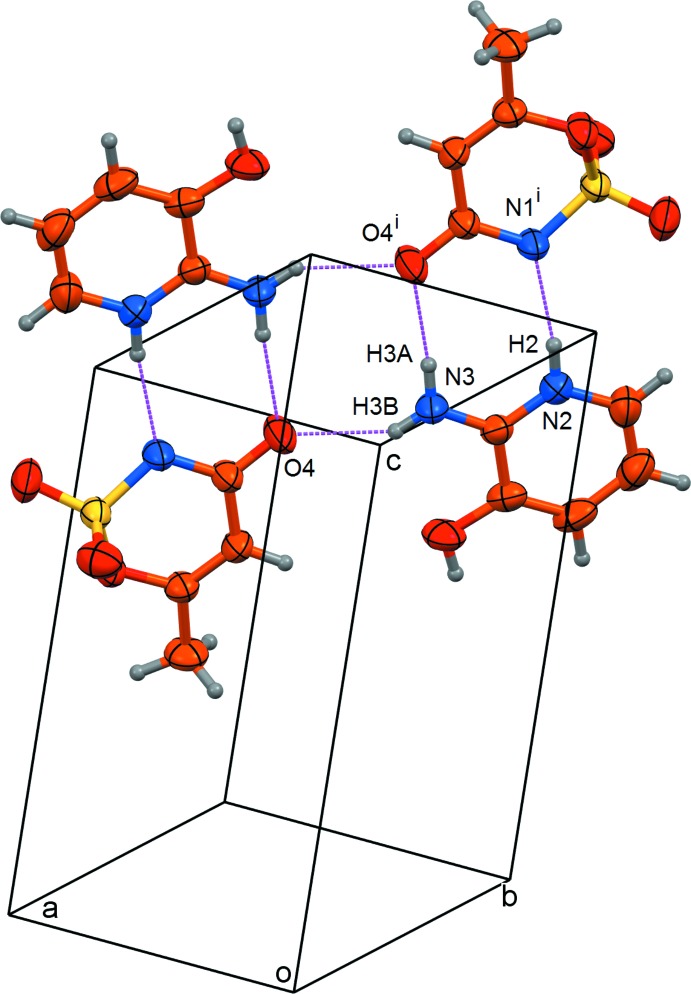
A view of the centrosymmetric aggregate formed by strong N—H⋯N and N—H⋯O hydrogen bonds (dashed lines). Displacement ellipsoids are drawn at the 50% probability level. Symmetry code: (i) 1 − *x*, 1 − *y*, 2 − *z*.

**Figure 3:**
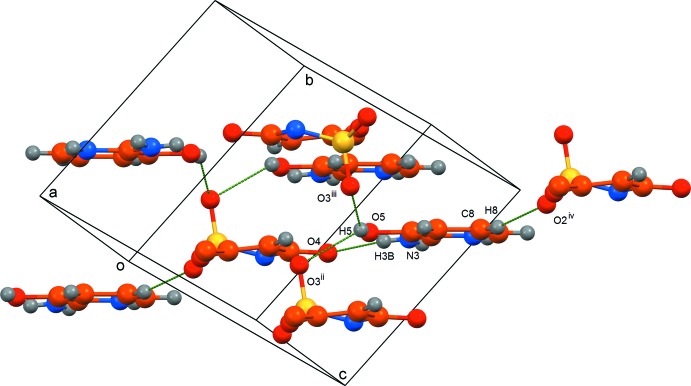
A view of the crystal packing of the title compound showing the three-dimensional system of hydrogen bonds. Methyl H atoms are omitted for clarity. Symmetry codes: (ii) *x* − 1, *y*, *z*; (iii) 1 − *x*, 1 − *y*, 1 − *z*; (iv) *x* − 2, *y* + 1, *z*.

The acesulfamate anions are linked to the 2-amino-3-hy­droxy­pyridinium cations by strong N—H⋯N and N—H⋯O hydrogen bonds, forming centrosymmetric aggregates each consisting of two cations and two anions (Table 2 ▸, Fig. 2 ▸). These aggregates are linked into a three-dimensional structure by weak O—H⋯O hydrogen bonds involving the sulfonyl groups and by C—H⋯O contacts (Table 2 ▸, Fig. 3 ▸). The shortest inter­centroid separation in (I) is only 4.1798 (15) Å, and thus the π-stacking inter­actions in this structure are insignificant.

## Database survey   

A search of the Cambridge Structural Database (CSD, version 5.39; Groom *et al.*, 2016 ▸) gave 54 hits for the oxa­thia­zin moiety. The compounds most closely related to (I) are 3-carbamoylpyridin-1-ium 6-methyl-2,2,4-trioxo-2*H*,4*H*-1,2,3-oxa­thia­zin-3-ide hemihydrate (CIHDEF; Wang *et al.*, 2018 ▸), 3-carb­oxy­pyridin-1-ium 6-methyl-2,2,4-trioxo-2*H*,4*H*-1,2,3-oxa­thia­zin-3-ide (CIHDIJ; Wang *et al.*, 2018 ▸), 6-amino-2-oxo-2,3-di­hydro­pyrimidin-1-ium 6-methyl-2,2,4-trioxo-2*H*,4*H*-1,2,3-oxa­thia­zin-3-ide 4-amino­pyrimidin-2(1*H*)-one (CIHFEH; Wang *et al.*, 2018 ▸), 5-fluoro-2-oxo-2,3-di­hydro­pyrimidin-4(1*H*)-iminium 6-methyl-4-oxo-4*H*-1,2,3-oxa­thia­zin-3-ide 2,2-dioxide hemihydrate (GONLIG; Wang *et al.*, 2014 ▸), potassium 6-methyl-1,2,3-oxa­thia­zin-4-one-2,2-dioxide (KMOTZD; Paulus 1975 ▸), thallium(I) 6-methyl-4-oxo-4*H*-1,2,3-oxa­thia­zin-3-ide 2,2-dioxide (OCAHUY; Baran *et al.*, 2015 ▸), choline acesulfamate (ODIHOZ; Nockemann *et al.*, 2007 ▸) and rubid­ium 6-methyl-4-oxo-4*H*-1,2,3-oxa­thia­zin-3-ide 2,2-dioxide (SURCIT; Piro *et al.*, 2015 ▸). In GONLIG, the molecules are linked by N—H⋯O hydrogen bonds, as in the title compound. In SURCIT, the carbonyl C=O bond distance is 1.231 (5) Å and the sulfoxide S=O bond lengthsare 1.415 (3) and 1.421 (3) Å, which are close toose in the title compound.

## Hirshfeld surface analysis   

**Figure 4:**
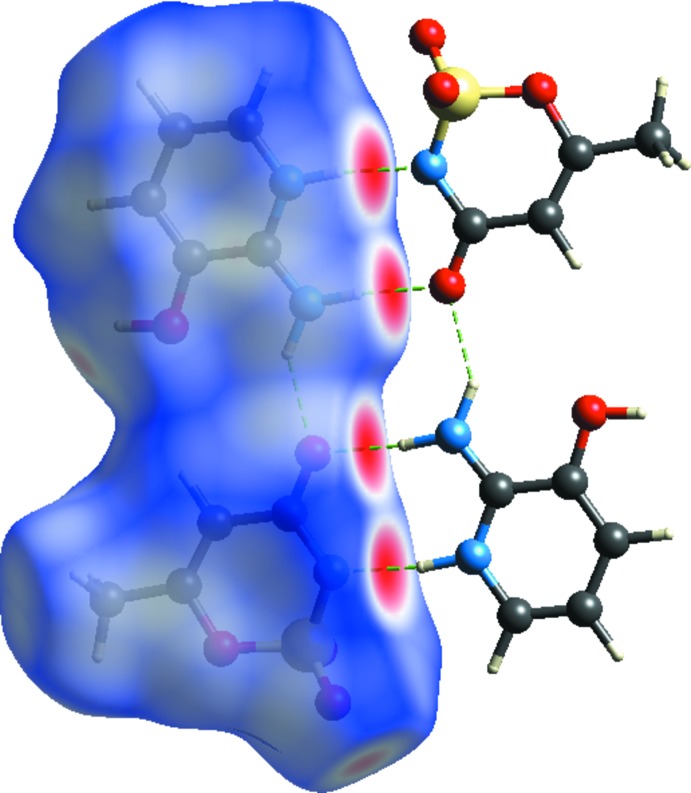
A view of the three-dimensional Hirshfeld surface of the title compound plotted over *d*_norm_ in the range −0.5966 to +1.0568 a.u.

**Figure 5:**
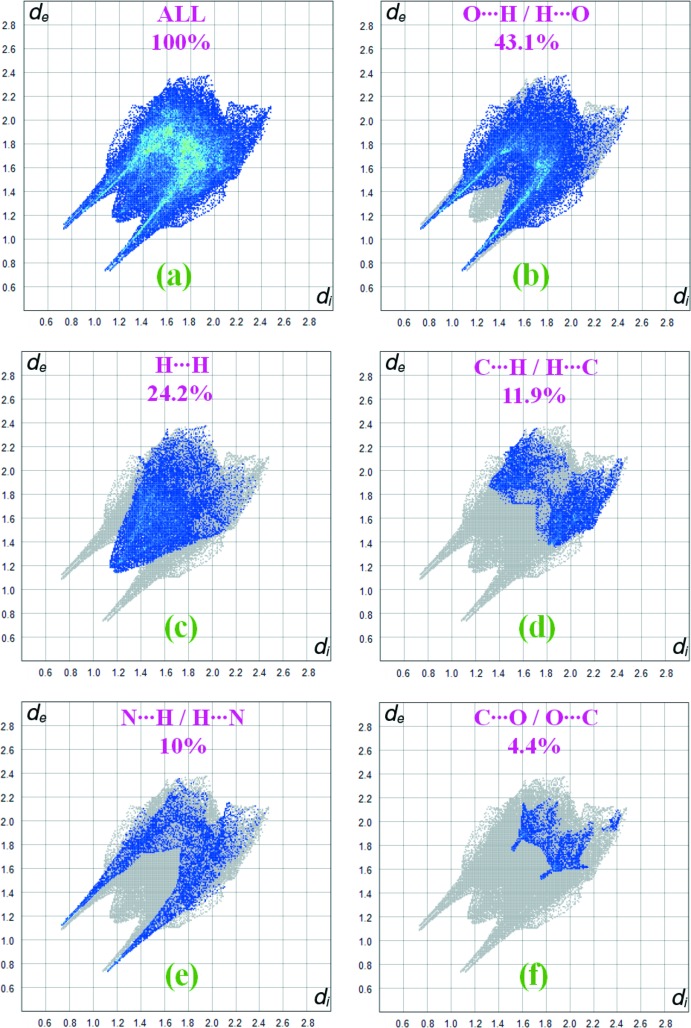
The two-dimensional fingerprint plots for the title compound showing (*a*) all inter­actions, (*b*) O⋯H/H⋯O, (*c*) H⋯H, (*d*) C⋯H/H⋯C, (*e*) N⋯H/H⋯N and (*f*) C⋯O/O⋯C inter­actions.

In order to visualize the inter­molecular inter­actions in the crystal of (I), Hirshfeld surface analysis (Spackman & Jayatilaka, 2009 ▸) was carried out using *CrystalExplorer17.5* (Turner *et al.*, 2017 ▸). Fig. 4 ▸ shows the Hirshfeld surface and the inter­molecular contacts of the title compound mapped over *d*_norm_ in the range −0.5966 to +1.0568 a.u. The red regions (distances shorter than the sum of the van der Waals radii) are apparent around the oxygen atom O4, which participates in the N—H⋯O contacts, and around the nitro­gen atom N1, which participates in the N—H⋯N contacts (Fig. 2 ▸, Table 2 ▸). The fingerprint plots for (I) are given in Fig. 5 ▸. The largest contribution to the overall crystal packing is from O⋯H/H⋯O inter­actions (43.1%). H⋯H contacts provide another significant contribution to the Hirshfeld surface of 24.2%. The N⋯H/H⋯N contacts appear as a pair of characteristic tips in the fingerprint plots; they contribute 10% to the Hirshfeld surface (Table 2 ▸).

## Synthesis and crystallization   

Potassium acesulfamate (1 mmol) was dissolved in 15 mL ethanol and heated to 348 K. To this solution 1 mmol of 2-amino-3-hy­droxy­pyridine in 15 mL of ethanol was added slowly under continuous stirring. After the addition, the solution was stirred for another 6 min at the same temperature. The compound thus formed was separated from the solution and then recrystallized from ethanol solution at room temperature. The red needle-shaped crystals obtained were filtered, washed with ethyl acetate and dried, yield 91%.

## Refinement   

Crystal data, data collection and structure refinement details are summarized in Table 3 ▸. All C-bound hydrogen atoms were placed in idealized positions and refined isotropically using a riding model, with *U*_iso_(H) = 1.5*U*_eq_(C) for methyl and with *U*_iso_(H) = 1.2*U*_eq_(C) for other C atoms, C—H = 0.96 Å for methyl and 0.93 Å for *sp* ^2^-hybridized C atoms. All other H atoms were located from the difference map and refined freely.

## Supplementary Material

Crystal structure: contains datablock(s) I. DOI: 10.1107/S2056989020003813/yk2127sup1.cif

Structure factors: contains datablock(s) I. DOI: 10.1107/S2056989020003813/yk2127Isup2.hkl

Supporting information file. DOI: 10.1107/S2056989020003813/yk2127Isup3.cml

CCDC reference: 1920140

Additional supporting information:  crystallographic information; 3D view; checkCIF report

## Figures and Tables

**Table 1:** Selected bond lengths (Å)

S1—O2	1.4149 (17)	O5—C6	1.353 (3)
S1—O3	1.4235 (18)	O4—C1	1.236 (3)
S1—N1	1.5605 (17)	N2—C5	1.336 (3)
S1—O1	1.6204 (15)	N3—C5	1.317 (3)
O1—C3	1.383 (2)	N1—C1	1.359 (3)

**Table 2:** Hydrogen-bond geometry (Å, °)

*D*—H⋯*A*	*D*—H	H⋯*A*	*D*⋯*A*	*D*—H⋯*A*
N3—H3*B*⋯O4	0.81 (3)	2.10 (3)	2.808 (3)	145 (2)
N3—H3*A*⋯O4^i^	0.85 (3)	2.00 (3)	2.846 (3)	175 (3)
N2—H2⋯N1^i^	0.90 (3)	1.99 (3)	2.871 (3)	168 (3)
O5—H5⋯O3^ii^	0.84 (4)	2.50 (4)	3.090 (2)	128 (3)
O5—H5⋯O3^iii^	0.84 (4)	2.25 (4)	2.995 (2)	147 (3)
C8—H8⋯O2^iv^	0.93	2.49	3.402 (3)	166

Symmetry codes: (i) 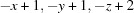; (ii) 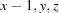; (iii) 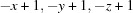; (iv) 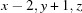.

**Table 3:** Experimental details

Crystal data	
Chemical formula	C_5_H_7_N_2_O^+^·C_4_H_4_NO_4_S^−^
*M*_r_	273.27
Crystal system, space group	Triclinic, *P* 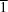
Temperature (K)	296
*a*, *b*, *c* (Å)	7.1676 (5), 9.1175 (7), 10.1554 (8)
α, β, γ (°)	66.174 (6), 80.225 (6), 71.803 (6)
*V* (Å^3^)	576.01 (8)
*Z*	2
Radiation type	Mo *K*α
μ (mm^−1^)	0.30
Crystal size (mm)	0.57 × 0.42 × 0.21

Data collection	
Diffractometer	STOE IPDS 2
Absorption correction	Integration (*X-RED32*; Stoe & Cie, 2002 ▸)
*T*_min_, *T*_max_	0.855, 0.953
No. of measured, independent and observed [*I* > 2σ(*I*)] reflections	5116, 2263, 1951
*R*_int_	0.031
(sin θ/λ)_max_ (Å^−1^)	0.617

Refinement	
*R*[*F* ^2^ > 2σ(*F* ^2^)], *wR*(*F* ^2^), *S*	0.040, 0.114, 1.08
No. of reflections	2263
No. of parameters	179
H-atom treatment	H atoms treated by a mixture of independent and constrained refinement
Δρ_max_, Δρ_min_ (e Å^−3^)	0.34, −0.26

Computer programs: *X-AREA* and *X-RED* (Stoe & Cie, 2002 ▸), *SHELXT2017* (Sheldrick, 2015*a* ▸), *SHELXL2017* (Sheldrick, 2015*b* ▸), *PLATON* (Spek, 2020 ▸) and *WinGX* (Farrugia, 2012 ▸).

## supplementary crystallographic information

### Crystal data

**Table:**

C_5_H_7_N_2_O^+^·C_4_H_4_NO_4_S^−^	*Z* = 2
*M**_r_* = 273.27	*F*(000) = 284
Triclinic, *P*1	*D*_x_ = 1.576 Mg m^−^^3^
*a* = 7.1676 (5) Å	Mo *K*α radiation, λ = 0.71073 Å
*b* = 9.1175 (7) Å	Cell parameters from 10423 reflections
*c* = 10.1554 (8) Å	θ = 3.0–31.5°
α = 66.174 (6)°	µ = 0.30 mm^−^^1^
β = 80.225 (6)°	*T* = 296 K
γ = 71.803 (6)°	Prism, red
*V* = 576.01 (8) Å^3^	0.57 × 0.42 × 0.21 mm

### Data collection

**Table:**

STOE IPDS 2 diffractometer	2263 independent reflections
Radiation source: sealed X-ray tube, 12 x 0.4 mm long-fine focus	1951 reflections with *I* > 2σ(*I*)
Detector resolution: 6.67 pixels mm^-1^	*R*_int_ = 0.031
rotation method scans	θ_max_ = 26.0°, θ_min_ = 3.0°
Absorption correction: integration (X-RED32; Stoe & Cie, 2002)	*h* = −8→8
*T*_min_ = 0.855, *T*_max_ = 0.953	*k* = −10→11
5116 measured reflections	*l* = −12→12

### Refinement

**Table:**

Refinement on *F*^2^	Primary atom site location: structure-invariant direct methods
Least-squares matrix: full	Secondary atom site location: difference Fourier map
*R*[*F*^2^ > 2σ(*F*^2^)] = 0.040	Hydrogen site location: mixed
*wR*(*F*^2^) = 0.114	H atoms treated by a mixture of independent and constrained refinement
*S* = 1.08	*w* = 1/[σ^2^(*F*_o_^2^) + (0.0615*P*)^2^ + 0.1608*P*] where *P* = (*F*_o_^2^ + 2*F*_c_^2^)/3
2263 reflections	(Δ/σ)_max_ = 0.001
179 parameters	Δρ_max_ = 0.34 e Å^−^^3^
0 restraints	Δρ_min_ = −0.26 e Å^−^^3^

### Special details

**Table:**

Geometry. All esds (except the esd in the dihedral angle between two l.s. planes) are estimated using the full covariance matrix. The cell esds are taken into account individually in the estimation of esds in distances, angles and torsion angles; correlations between esds in cell parameters are only used when they are defined by crystal symmetry. An approximate (isotropic) treatment of cell esds is used for estimating esds involving l.s. planes.

### Fractional atomic coordinates and isotropic or equivalent isotropic displacement parameters (Å^2^)

**Table:**

	*x*	*y*	*z*	*U*_iso_*/*U*_eq_	
S1	1.03944 (7)	0.17969 (7)	0.69020 (6)	0.04075 (18)	
O1	0.9160 (2)	0.10735 (19)	0.62259 (17)	0.0478 (4)	
O3	1.0909 (3)	0.3136 (2)	0.57109 (18)	0.0601 (5)	
O5	0.1101 (3)	0.5582 (2)	0.70493 (17)	0.0553 (4)	
O4	0.5989 (2)	0.3846 (3)	0.8548 (2)	0.0666 (5)	
N2	−0.0570 (3)	0.7569 (2)	0.9560 (2)	0.0438 (4)	
O2	1.1970 (2)	0.0414 (2)	0.7566 (2)	0.0659 (5)	
N3	0.2510 (3)	0.6079 (3)	0.9066 (2)	0.0455 (4)	
N1	0.8981 (2)	0.2371 (2)	0.80563 (19)	0.0451 (4)	
C5	0.0626 (3)	0.6702 (2)	0.8807 (2)	0.0371 (4)	
C3	0.7205 (3)	0.1932 (3)	0.5985 (2)	0.0407 (4)	
C1	0.7049 (3)	0.3113 (3)	0.7784 (2)	0.0441 (5)	
C6	−0.0206 (3)	0.6490 (3)	0.7748 (2)	0.0435 (5)	
C2	0.6223 (3)	0.2932 (3)	0.6663 (2)	0.0461 (5)	
H2A	0.494302	0.354995	0.641245	0.055*	
C7	−0.2159 (4)	0.7151 (3)	0.7551 (2)	0.0565 (6)	
H7	−0.271871	0.700166	0.686842	0.068*	
C9	−0.2527 (3)	0.8245 (3)	0.9352 (3)	0.0564 (6)	
H9	−0.330076	0.884168	0.990109	0.068*	
C8	−0.3342 (3)	0.8056 (4)	0.8363 (3)	0.0636 (7)	
H8	−0.467976	0.852033	0.821546	0.076*	
C4	0.6425 (4)	0.1525 (4)	0.4958 (3)	0.0565 (6)	
H4A	0.745607	0.076688	0.462632	0.085*	
H4B	0.537932	0.101580	0.542857	0.085*	
H4C	0.593203	0.252581	0.415180	0.085*	
H3A	0.289 (4)	0.614 (3)	0.978 (3)	0.054 (7)*	
H3B	0.320 (4)	0.547 (3)	0.867 (3)	0.053 (7)*	
H5	0.048 (6)	0.554 (5)	0.644 (4)	0.105 (12)*	
H2	−0.014 (4)	0.775 (4)	1.024 (3)	0.066 (8)*	

### Atomic displacement parameters (Å^2^)

**Table:**

	*U*^11^	*U*^22^	*U*^33^	*U*^12^	*U*^13^	*U*^23^
S1	0.0320 (3)	0.0515 (3)	0.0452 (3)	−0.0048 (2)	−0.00460 (19)	−0.0282 (2)
O1	0.0402 (8)	0.0550 (9)	0.0611 (9)	−0.0052 (7)	−0.0055 (7)	−0.0393 (8)
O3	0.0616 (10)	0.0769 (12)	0.0537 (9)	−0.0322 (9)	0.0023 (8)	−0.0281 (9)
O5	0.0630 (10)	0.0730 (11)	0.0459 (9)	−0.0222 (9)	−0.0056 (8)	−0.0342 (8)
O4	0.0420 (8)	0.1016 (14)	0.0750 (11)	0.0038 (9)	−0.0074 (8)	−0.0674 (11)
N2	0.0387 (9)	0.0502 (10)	0.0425 (9)	−0.0055 (8)	−0.0056 (7)	−0.0209 (8)
O2	0.0425 (9)	0.0720 (12)	0.0812 (12)	0.0127 (8)	−0.0196 (8)	−0.0414 (10)
N3	0.0394 (9)	0.0572 (11)	0.0463 (10)	−0.0011 (8)	−0.0112 (8)	−0.0312 (9)
N1	0.0343 (8)	0.0645 (11)	0.0460 (9)	−0.0050 (8)	−0.0062 (7)	−0.0349 (9)
C5	0.0383 (10)	0.0395 (10)	0.0325 (9)	−0.0097 (8)	−0.0047 (7)	−0.0118 (8)
C3	0.0386 (10)	0.0495 (11)	0.0404 (10)	−0.0119 (9)	−0.0054 (8)	−0.0218 (9)
C1	0.0363 (10)	0.0569 (12)	0.0459 (11)	−0.0052 (9)	−0.0032 (8)	−0.0311 (10)
C6	0.0492 (11)	0.0508 (12)	0.0328 (9)	−0.0200 (9)	−0.0067 (8)	−0.0113 (9)
C2	0.0338 (10)	0.0596 (13)	0.0481 (11)	−0.0017 (9)	−0.0102 (9)	−0.0283 (10)
C7	0.0522 (13)	0.0729 (16)	0.0446 (12)	−0.0244 (12)	−0.0171 (10)	−0.0109 (12)
C9	0.0389 (11)	0.0605 (14)	0.0590 (14)	−0.0012 (10)	−0.0020 (10)	−0.0209 (12)
C8	0.0370 (12)	0.0758 (17)	0.0612 (15)	−0.0102 (11)	−0.0122 (11)	−0.0089 (13)
C4	0.0589 (14)	0.0759 (16)	0.0524 (13)	−0.0233 (12)	−0.0076 (11)	−0.0361 (12)

### Geometric parameters (Å, º)

**Table:**

S1—O2	1.4149 (17)	C5—C6	1.417 (3)
S1—O3	1.4235 (18)	C3—C2	1.320 (3)
S1—N1	1.5605 (17)	C3—C4	1.476 (3)
S1—O1	1.6204 (15)	C1—C2	1.454 (3)
O1—C3	1.383 (2)	C6—C7	1.354 (3)
O5—C6	1.353 (3)	C2—H2A	0.9300
O5—H5	0.84 (4)	C7—C8	1.398 (4)
O4—C1	1.236 (3)	C7—H7	0.9300
N2—C5	1.336 (3)	C9—C8	1.336 (4)
N2—C9	1.359 (3)	C9—H9	0.9300
N2—H2	0.90 (3)	C8—H8	0.9300
N3—C5	1.317 (3)	C4—H4A	0.9600
N3—H3A	0.85 (3)	C4—H4B	0.9600
N3—H3B	0.81 (3)	C4—H4C	0.9600
N1—C1	1.359 (3)		
			
O2—S1—O3	116.38 (12)	N1—C1—C2	119.43 (18)
O2—S1—N1	110.55 (11)	O5—C6—C7	126.8 (2)
O3—S1—N1	113.19 (11)	O5—C6—C5	113.93 (18)
O2—S1—O1	104.59 (10)	C7—C6—C5	119.2 (2)
O3—S1—O1	104.86 (9)	C3—C2—C1	123.12 (19)
N1—S1—O1	106.18 (9)	C3—C2—H2A	118.4
C3—O1—S1	116.85 (13)	C1—C2—H2A	118.4
C6—O5—H5	107 (3)	C6—C7—C8	120.7 (2)
C5—N2—C9	123.0 (2)	C6—C7—H7	119.7
C5—N2—H2	122.3 (18)	C8—C7—H7	119.7
C9—N2—H2	114.6 (18)	C8—C9—N2	120.2 (2)
C5—N3—H3A	117.7 (18)	C8—C9—H9	119.9
C5—N3—H3B	118.7 (19)	N2—C9—H9	119.9
H3A—N3—H3B	122 (3)	C9—C8—C7	119.2 (2)
C1—N1—S1	118.98 (14)	C9—C8—H8	120.4
N3—C5—N2	119.84 (18)	C7—C8—H8	120.4
N3—C5—C6	122.40 (19)	C3—C4—H4A	109.5
N2—C5—C6	117.77 (18)	C3—C4—H4B	109.5
C2—C3—O1	121.46 (18)	H4A—C4—H4B	109.5
C2—C3—C4	126.9 (2)	C3—C4—H4C	109.5
O1—C3—C4	111.62 (18)	H4A—C4—H4C	109.5
O4—C1—N1	119.67 (18)	H4B—C4—H4C	109.5
O4—C1—C2	120.74 (19)		
			
O2—S1—O1—C3	156.84 (16)	N2—C5—C6—O5	−179.25 (18)
O3—S1—O1—C3	−80.20 (17)	N3—C5—C6—C7	179.3 (2)
N1—S1—O1—C3	39.88 (17)	N2—C5—C6—C7	−0.6 (3)
O2—S1—N1—C1	−151.14 (19)	O1—C3—C2—C1	−4.9 (4)
O3—S1—N1—C1	76.3 (2)	C4—C3—C2—C1	172.6 (2)
O1—S1—N1—C1	−38.2 (2)	O4—C1—C2—C3	−168.2 (2)
C9—N2—C5—N3	−179.9 (2)	N1—C1—C2—C3	7.4 (4)
C9—N2—C5—C6	0.0 (3)	O5—C6—C7—C8	179.5 (2)
S1—O1—C3—C2	−20.9 (3)	C5—C6—C7—C8	1.0 (4)
S1—O1—C3—C4	161.22 (16)	C5—N2—C9—C8	0.2 (4)
S1—N1—C1—O4	−166.63 (19)	N2—C9—C8—C7	0.2 (4)
S1—N1—C1—C2	17.7 (3)	C6—C7—C8—C9	−0.8 (4)
N3—C5—C6—O5	0.7 (3)		

### Hydrogen-bond geometry (Å, º)

**Table:**

*D*—H···*A*	*D*—H	H···*A*	*D*···*A*	*D*—H···*A*
N3—H3*B*···O4	0.81 (3)	2.10 (3)	2.808 (3)	145 (2)
N3—H3*A*···O4^i^	0.85 (3)	2.00 (3)	2.846 (3)	175 (3)
N2—H2···N1^i^	0.90 (3)	1.99 (3)	2.871 (3)	168 (3)
O5—H5···O3^ii^	0.84 (4)	2.50 (4)	3.090 (2)	128 (3)
O5—H5···O3^iii^	0.84 (4)	2.25 (4)	2.995 (2)	147 (3)
C8—H8···O2^iv^	0.93	2.49	3.402 (3)	166

Symmetry codes: (i) −*x*+1, −*y*+1, −*z*+2; (ii) *x*−1, *y*, *z*; (iii) −*x*+1, −*y*+1, −*z*+1; (iv) *x*−2, *y*+1, *z*.
